# Healthy Aging Metabolomic and Proteomic Signatures Across Multiple Physiological Compartments

**DOI:** 10.1111/acel.70014

**Published:** 2025-02-14

**Authors:** R. Moaddel, J. Candia, C. Ubaida‐Mohien, T. Tanaka, A. Z. Moore, M. Zhu, G. Fantoni, S. Church, J. D'Agostino, J. Fan, N. Shehadeh, S. De, E. Lehrmann, M. Kaileh, E. Simonsick, R. Sen, J. M. Egan, L. Ferrucci

**Affiliations:** ^1^ Biomedical Research Centre National Institute on Aging, NIH Baltimore Maryland USA

**Keywords:** accelerated aging, aging, biological aging, elastic net, extracellular matrix, inflammation, mitochondrial health, plasma proteomics, senescence, urine proteomics

## Abstract

The study of biomarkers in biofluids and tissues expanded our understanding of the biological processes that drive physiological and functional manifestations of aging. However, most of these studies were limited to examining one biological compartment, an approach that fails to recognize that aging pervasively affects the whole body. The simultaneous modeling of hundreds of metabolites and proteins across multiple compartments may provide a more detailed picture of healthy aging and point to differences between chronological and biological aging. Herein, we report proteomic analyses of plasma and urine collected in healthy men and women, age 22–92 years. Using these data, we developed a series of metabolomic and proteomic predictors of chronological age for plasma, urine, and skeletal muscle. We then defined a biological aging score, which measures the departure between an individual's predicted age and the expected predicted age for that individual based on the full cohort. We show that these predictors are significantly and independently related to clinical phenotypes important for aging, such as inflammation, iron deficiency anemia, muscle mass, and renal and hepatic functions. Despite a different set of selected biomarkers in each compartment, the different scores reflect a similar degree of deviation from healthy aging in single individuals, thus allowing identification of subjects with significant accelerated or decelerated biological aging.

## Graphical abstract

The association of the aging scores with the clinical covariates suggests increased inflammation, strong predisposition to iron deficiency anemia, decline in muscle mass, and renal and hepatic functions with accelerated aging.

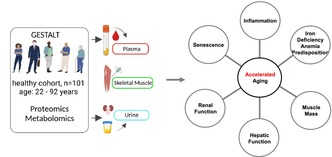

## 1 Introduction

Chronological aging is associated with a continuum of bodily changes that in the early years of life build/confer physiologic reserve and resilience and then progress through to the slow deterioration of underlying structural components and loss of physiological integrity that tends to accelerate in late life. Human studies have investigated age‐related biomarkers in biofluids and tissues, such as blood, urine, cerebrospinal fluid, and skeletal muscle, in an attempt to infer biological changes that occur with aging and drive the typical phenotypes of aging, independent of the underlying disease (Johnson et al. 2019; Teruya et al. 2020; Ubaida‐Mohien et al. 2019). As changes that occur across compartments are, of necessity, interconnected, the simultaneous analysis of multiple compartments may provide a deeper understanding of these mechanisms (Moaddel et al. 2021). Recently, we investigated age‐related differences in the metabolome in three compartments—plasma, muscle, and urine—to shed light on the underlying mechanisms that drive age‐related changes in participants who were healthy based on a comprehensive clinical evaluation performed by trained health professionals (Moaddel et al. 2023). We found that inflammation and cellular senescence, microbial metabolism, mitochondrial health, sphingolipid metabolism, lysosomal membrane permeabilization, and vascular and renal functions were the main biological pathways systematically compromised with age (Moaddel et al. 2023). Notably, the rate of change of age‐related mechanisms varies across individuals, and this heterogeneity is reflective of the discrepancy between chronological and biological aging at the population level.

The potential for developing surrogate measures of biological age was initially demonstrated using DNA methylation data gathered from blood and other tissues (Hannum et al. 2013; Horvath 2013). There is evidence that DNA methylation modulates gene expression that, in turn, impacts protein synthesis and turnover (Hannum et al. 2013; Horvath and Raj 2018). The recent development of plasma proteomic signatures of biological age further confirms the notion that biological changes with age are reflected in circulating proteins (Johnson et al. 2021; Tanaka et al. 2018). Adding to such complexity, different compartments, including different organs, for example, the liver and kidney and different systems including immune and metabolic systems, appear to age at different rates, resulting in compartment‐dependent “clocks” (Nie et al. 2022).

We hypothesized that the integration of information on proteomic and metabolomic data as well as clinical covariates in a cohort of generally healthy individuals representing a broad age range, such as the one described herein, should allow for the identification of “aging scores” (defined as the difference between an individual's estimated biological age and their chronological age) that are relatively independent of disease. Here, we first identified proteins that were cross‐sectionally associated at baseline with age in plasma and urine, and we then simultaneously modeled plasma, muscle and urine metabolomic (Moaddel et al. 2023), muscle proteomic (Ubaida‐Mohien et al. 2019), and plasma and urine proteomic data at baseline to provide mechanistic information so as to generate compartment‐specific molecular signatures of aging. Finally, we correlated the compartment data with clinical measures that represent the phenotypic manifestations of such molecular signatures.

## 2 Results

### 2.1 Analysis of Proteins in Plasma and Urine

**FIGURE 1:**
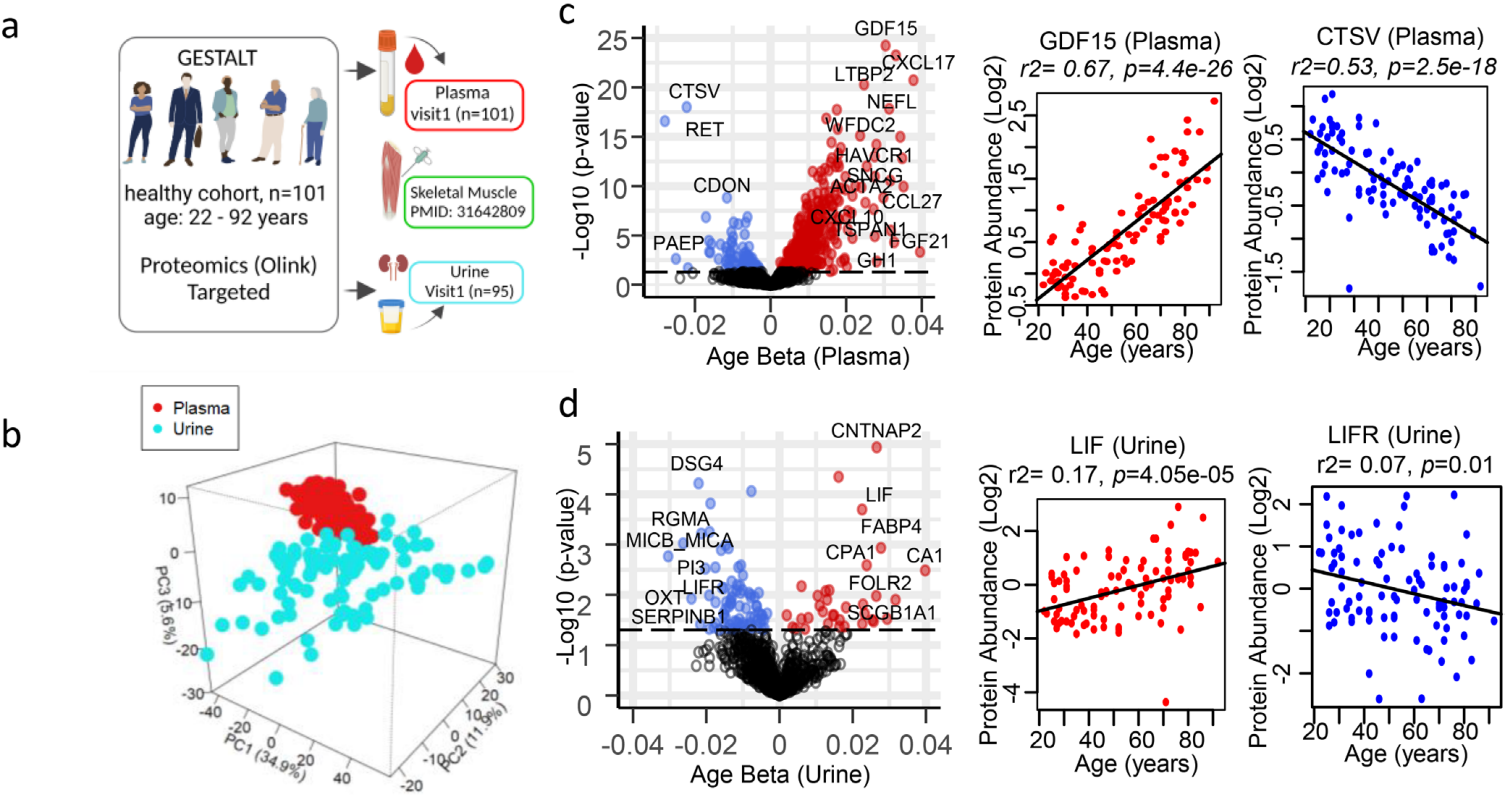
Analysis of healthy human aging in plasma and urine. (a) Overview of the study design. (b) Principal component analysis of all proteins quantified from participants from plasma (red) and urine (blue). (c) Volcano plot of all plasma proteins quantified from 101 GESTALT participants, age‐range 22–92 years at baseline (visit 1). Protein significance with age is plotted on the y‐axis, protein‐age association is plotted on x‐axis, and positive age effect size shows proteins association higher in older age and negative age effect size shows association lower in older age. Top age‐associated proteins are shown GDF15 (over‐represented) and CTSV (under‐represented) (d) Volcano plot of all urine proteins quantified from 95 GESTALT participants. Protein significance with age is plotted on the y‐axis, protein‐age association is plotted on x‐axis, and positive age effect size shows proteins association higher in older age and negative age effect size shows association lower in older age. Over‐represented protein LIF and under‐represented protein LIFR is shown.

**FIGURE 2:**
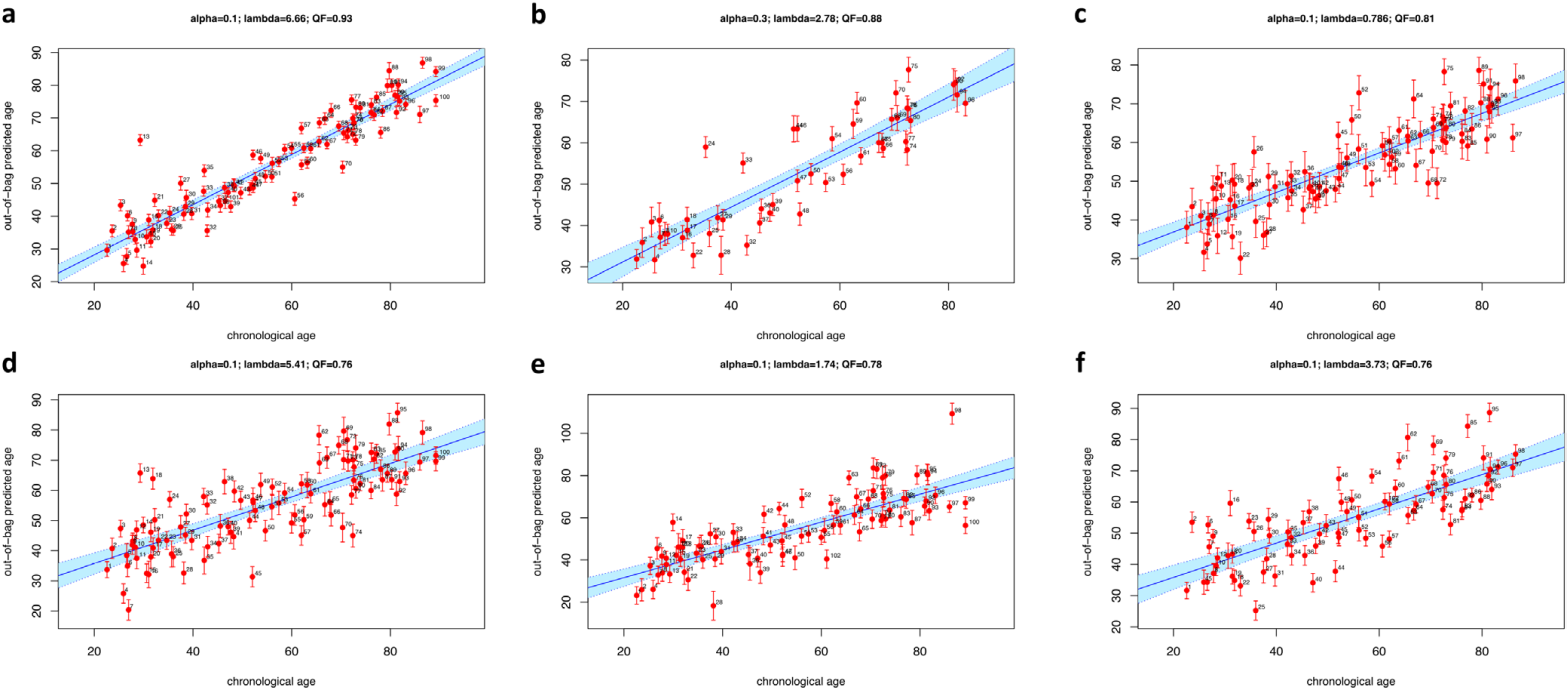
Prediction of chronological age from cross‐validated elastic net models. For each compartment/omics, predicted chronological age (y‐axis) is compared against the true chronological age as response variable (x‐axis). Elastic net models were assessed using eNetXplorer to generate 500 runs of fivefold cross‐validation; the mixing parameter alpha and penalty parameter lambda used in each case are shown, as well as Pearson's correlation as quality factor (QF) used to select the best‐performing model. (a) Plasma proteomics; (b) Muscle proteomics; (c) Urine proteomics; (d) Plasma metabolomics; (e) Muscle metabolomics; (f) Urine metabolomics.

We conducted discovery plasma and urine proteomic studies using the Explore 1536 Olink (proximity extension assay (PEA)) in healthy men and women from the Genetic and Epigenetic Study of Aging Laboratory Testing (GESTALT, age‐range 22–92 years) study (Tanaka et al. 2018) at baseline (*n* = 101; Figure 1a) and 2‐year follow‐up (*n* = 65). Participants in GESTALT at the time of enrollment were free of chronic conditions including cardiovascular, pulmonary, gastrointestinal, autoimmune, and metabolic diseases, did not smoke cigarettes, and did not have active cancer in the preceding 10 years. Demographic characteristics of the study participants are summarized in Table 1. In this study, we carried out the cross‐sectional analysis of the measured proteins in both plasma and urine compartments at baseline and the 2‐year follow‐up. Relative concentrations of 1432 and 1055 proteins were detected in plasma and urine at baseline, respectively. Plasma and urine proteins showed clear separation between compartments, with the first three principal components explaining 52.4% of the variance between compartments at baseline (Figure 1b). Linear regression models adjusted for sex, race, and BMI were performed to identify proteins that were significantly associated with age in plasma and urine at baseline (Figure 1c,d) and at the 2‐year follow‐up. As seen in Figure 1c, the majority of age‐associated proteins in plasma were over‐represented (increasing with age), while the majority of age‐associated proteins in urine were under‐represented (decreasing with age), with age (Figure 1d). A significant correlation was observed between the age *β* coefficients at baseline and 2‐year follow‐up for the age‐associated proteins, as well as for all proteins in both plasma and urine (*r* ^2^ = 0.78, *p* = < 2e‐16; and *r* ^2^ = 0.45, *p* = 4.83e‐140, respectively; Figure S1). We also carried out cross‐validated elastic net regression models using proteomic and metabolomic data across multiple compartments (plasma, muscle, and urine) to create a multivariate signature for each compartment/−omic as a predictor of chronological age. The use of a healthy aging cohort, such as the one described herein, in combination with the integration of proteomic and metabolomic data and clinical covariates, allows for the identification of biomarkers that deviate from chronological age, per se independent of disease, as much as is possible, which may allow a more detailed picture of healthy aging that may enable the systematic study of differences resulting from chronological and biological aging within and between compartments.

**TABLE 1:** Demographics of the participants of the Genetic and Epigenetic Study of Aging Laboratory Testing (GESTALT, age‐range 22–92 years) in plasma and urine.

Matrix	Phenotype				Age groups (*n*)				
	*n*	BMI	Sex (Male%)	Race (White%)	20–34	35–49	50–64	65–79	80+
Plasma	101	25.6	55%	82%	23	20	18	28	12
Urine	95	25.6	53%	81%	21	21	18	25	10

### 2.2 Plasma Proteome Associated With Age

Of the 1432 plasma proteins that we detected, 475 (390 over‐represented and 85 under‐represented) were age‐associated at baseline in the cross‐sectional analysis (Figure 1c; Table S1) and 396 (332 over‐represented; 64 under‐represented) were age‐associated at the 2‐year follow‐up (Table S1). A list of the top 20 over‐ and under‐represented proteins and their functions is reported in Table S2, and the top significant over‐ and under‐represented proteins are shown in Figure 1c (right). 340 of these proteins were shared between baseline and the 2‐year follow‐up analysis (Table S3A).

In line to previous studies (Moaddel et al. 2021; Johnson et al. 2021; Tanaka et al. 2018), the top age‐associated proteins were related to inflammation and/or cellular senescence (Figure 1c). For example, GDF15, a member of the transforming growth factor‐β (TGF‐β) cytokine superfamily and a core member of the senescence‐associated secretory phenotype (SASP) proteins (Tanaka et al. 2018), was the top age‐associated protein in plasma (Figure 1c, right). CXCL17, also a core member of the SASP (Tanaka et al. 2018), CXCL9, a master biomarker of inflammation (Sayed et al. 2021), NEFL, a biomarker of neurodegeneration (Narayanan et al. 2021), and EDA2R, an inflammatory biomarker of aging across multiple tissues, were highly age‐associated (Barbera et al. 2022). Consistent with previous studies (Johnson et al. 2021), two of our top three under‐represented age‐associated proteins, RET, a receptor tyrosine kinase, and CDON, a hedgehog co‐receptor, were strongly associated with age. Several of the top under‐represented, age‐associated proteins are also related to inflammation. EGFR, another tyrosine kinase receptor (Romano and Bucci 2020), is regulated by the FOXO transcription factors that are well known to be involved with aging (Moaddel et al. 2021) and is associated with inflammation and senescence (Shang et al. 2020), and CDON has been found to be upregulated in endothelial cells treated with inflammatory cytokines (Chapouly et al. 2020). Consistent with these findings, STRING enrichment analysis of 390 over‐represented (Figure S2a) and 85 under‐represented age‐related proteins (Figure S2b) also identified inflammation‐related pathways, with 146 over‐represented proteins associated with immune system processes (Figure S2c), including several chemokine ligands (CCL2, 3, 11, 13, 14, 15, 16, 18, 23, 25, and CCL 27; CXCL8, 9, 10, 14, 16, and CXCL17) and several cytokines, including IL6 and IFNG and 24 of the under‐represented proteins. The over‐representation of cell adhesion molecules (CAMs; ICAM1, VCAM1, as well as ALCAM, BCAM, and MCAM) and a junctional adhesion molecule (JAM2) (Hintermann et al. 2018) also suggests increased inflammation. These findings illustrate that the modulation of the pro‐inflammatory state with age is complex and involves both pro‐ and anti‐inflammatory pathways, with the pro‐state dominating. As noted above, several of the top age‐associated proteins were SASP members (Table S4), with a total of 85 SASP proteins identified from the SASPAtlas (Basisty et al. 2020). In addition to these proteins, we identified several SASP proteins not in the SASPAtlas, including CXCL17, CCL3, CXCL9, FAS, MMP12, TNFRSF1A, TNFRSF1B, CDCP1, LY6D (Nagano et al. 2021), ACTA2 (Schafer et al. 2016), and CLEC5A. The identification of many SASP proteins is consistent with previous findings from plasma (Tanaka et al. 2018). Further, in a previous study using an untargeted LC–MS‐based proteomic method, 445 SASP proteins (Table S4) were identified in muscle in the same subjects (Ubaida‐Mohien et al. 2019), with the larger number in muscle likely resulting from the SASPAtlas and the skeletal muscle proteomics having been carried out by LC–MS‐based proteomics. The age‐associated increase of SASP proteins and cellular senescence markers (Franceschi et al. 2018; Schroth et al. 2020) would be expected to result in a greater secretion of growth factors. Accordingly, 78% (31/40) (Table S5) of the age‐associated growth factors identified in this study increased with age. Notable networks identified in the STRING network analysis included the increase of fibroblast growth factors (FGF5, 21 and 23) and IGF‐binding proteins (IGFBP1, 4, and 6; Figure S2). Growth factors that declined with age included IGFBP3, EGFR, and MEGF9. EGFR, as mentioned above, is a tyrosine kinase receptor that plays a role, along with IGF1 and insulin, in growth, differentiation, maintenance and repair of tissues and organs (Romano and Bucci 2020; Enwere et al. 2004), and MEGF9 contains multiple EGF‐like repeats and is involved in cell‐adhesion, receptor–ligand interactions, and tissue repair (Brandt‐Bohne et al. 2007). Lower values of IGFBP3 with older age is consistent with greater senescence (Hong and Kim 2018) as a decline in IGFBP3 results, in most cases, in increased free IGF1 and subsequent activation of its cognate receptor, IGF1R, which is also over‐represented with older age in our study, and would be predicted to result in the upregulation of the PI3K/Akt/mTOR signaling pathway (Hong and Kim 2018): IGF‐1/IGF‐1R activation is also modulated by extracellular matrix (ECM) proteins and integrins (Chen et al. 2020a).

Many of the top plasma proteome age‐associated proteins included ECM and ECM‐related proteins (respectively 78 and 102 proteins (Table S6)). Several galectins (LGALS1, 3, 4, and 9) that remodel the ECM in vitro (Dvoránková et al. 2011) and several cathepsins (CTSs; CTSV, CTSB, CTSL, CTSF, and CSTZ) which degrade the components of the ECM (Yadati et al. 2020), were age‐associated. CTSV, which participates in the proteolytic processing of a variety of ECM components (McCabe et al. 2020), was the only CTS to be under‐represented with age. IFNG has been reported to induce lysosomal membrane permeabilization resulting in the release of CTSs (Yadati et al. 2020) and was over‐represented with age in our study. Several angiopoietin‐related proteins (ANGPTLs; ANGPTL1, 2, 3, 4, and 7), which interact /associate with ECMs and are involved in multiple biological processes (lipid and glucose metabolism, inflammation, cancer, and, as their name suggests, hematopoiesis), were likewise over‐represented with age in plasma (Thorin et al. 2023). Several collagen species, representing the most abundant and diverse ECM molecules (Arseni et al. 2018), tended to be higher at older chronological age; however, this trend was statistically significant only for COL6A3 and COL18A1. Of note, zinc‐dependent endopeptidases Matrix metalloproteinases (MMPs 7 and 12), a Disintegrin and Metalloproteinase (ADAM 22 and 23) and a Disintegrin and Metalloproteinase with Thrombospondin Motif (ADAMTS13 and 16), were higher at older ages. These proteins, like CTSs, also degrade the ECM. COMP, an ECM glycoprotein (Posey et al. 2018), and LTBP2, an ECM protein that plays a role in cell adhesion (Shi et al. 2021), were also over‐represented with age in plasma. In the STRING network analysis of the plasma proteome (Figure S2a), Integrin alpha's V and 5 (ITGAV, ITGA5) and Beta's 1, 2, and 5 (ITGB1, 2 and 5) were major hubs in the over‐represented proteins; integrins are the main cell adhesion receptors of the ECM. The differences in integrins, CAMs, and ECM components with older age are consistent with the notion of integrins as the main mechano‐transducers connecting the actin cytoskeleton to the matrix. Further, these changes may suggest a stiffening of ECM with age (Ojha et al. 2022), which could result in increased ECM accumulation, mild hypoxia, and greater organ dysfunction in older persons (Selman and Pardo 2021). The changes in proteins described above suggest increased inflammation and cellular senescence that establish profound changes in the intercellular matrix and mitochondrial dysfunction with age (Selman and Pardo 2021).

The under‐representation of pyruvate kinase L/R (PKLR) as well as the nucleoside‐diphosphate kinase (NME3), SOD2, and the protein subunit of mitochondrial RNase P (RPM2) also points to mitochondrial dysfunction with age. RPM2 plays a role in the translation of cytochrome c oxidase, and the lack of RPM2 in cells results in accumulated lesions in their mitochondrial DNA (Stribinskis et al. 2001). NME3 is critical for the maintenance and function of the mitochondrial tubular network, and its loss leads to mitochondrial fragmentation and increased mitochondrial superoxide formation (Chen et al. 2020b). PKLR, the last step of the glycolytic pathway, is a regulator of lipid metabolism and mitochondrial function and potentially has a regulatory role in maintaining mitochondrial activity (Liu et al. 2019). The age‐associated decline of PKLR, which converts PEP to pyruvate, suggests the decreased availability of pyruvate for the TCA cycle (Liu et al. 2019). The overall results demonstrate global derangement of mitochondrial integrity and function with aging.

### 2.3 Urine Proteome Associated With Age

Of the 1055 proteins measured in urine, 113 were age‐associated (34 over‐represented; 79 under‐represented) in the cross‐sectional analysis at baseline (Figure 1d; Table S7) and 54 (17 over‐represented; 37 under‐represented) were age‐associated at the 2‐year follow‐up. Only 20 age‐associated proteins (5 over‐represented; 15 under‐represented) were shared between baseline and the 2‐year follow‐up analysis. We also performed normalization of the urinary proteomic data with urinary Cystatin C, a glomerular filtration marker that is not affected by age (Figure S3; Moaddel et al. 2023), to account for urine dilution. Our data demonstrated that the identified age‐associated proteins were similar (Table S8; Figure S3).

In agreement with our data in the plasma proteome, several of the top age‐associated proteins in urine were related to inflammation (Table S2). LIF, which plays a role in inflammatory renal diseases (Morel et al. 2000), was one of the top over‐represented proteins in urine. Interestingly, its receptor, the LIF Receptor (LIFR), was lower with age. The binding of LIF to LIFR leads to the activation of JAK/STAT and downstream MAPK cascades (Morel et al. 2000). Another critical mediator of the inflammatory process, the fatty acid binding protein FABP4, which plays an important role in obesity‐related metabolic diseases (Shrestha et al. 2018), was also over‐represented in urine and plasma. STRING network analysis of the age‐associated proteins in urine identified proteins related to the immune system including cytokines and/or cytokine signaling pathways, integrin (ITGB6), and several immune‐related pathways. Interestingly, none of the age‐associated plasma integrins were age‐associated in urine. Only ITGB6 (under‐represented) which increases integrin–ligand expression, macrophage infiltration, proinflammatory cytokine secretion, and signal transducer and activator of transcription 1 (STAT1) (Pang et al. 2023) was age‐associated in urine. CD274, one of the top under‐represented proteins in urine, involved in the CD274/PDCD1 pathway, serves as a negative regulator of T cells by preventing the overstimulation of physiological immune responses and preventing the loss of self‐tolerance (Ahtiainen et al. 2019). Several proteins in this same pathway (CD274, PDCD1, and PDCD1LG2) also were under‐represented with age. Interestingly, none of these proteins were age‐associated in plasma, suggesting that the mechanisms that cause their differential expression with aging are renal‐specific. KIRREL2, a member of the immunoglobulin superfamily of CAMs with β‐cell‐specific expression in humans (Yesildag et al. 2015), was under‐represented with aging in urine. This finding is also consistent with age‐related, pro‐inflammatory changes, which are associated with increased glomerular protein permeability. The age‐associated increase of IL17A, LIF, and MMP3 in urine also reflects an increase in the pro‐inflammatory state (Morel et al. 2000; Liang et al. 2021; Zenobia and Hajishengallis 2015). IL17 links T‐cell activation to neutrophil mobilization and activation (Zenobia and Hajishengallis 2015), and MMP3 plays a role in several processes including angiogenesis, cell growth, and cell invasion (Liang et al. 2021) and has also been identified as a SASP protein (Basisty et al. 2020). While a larger number of SASP proteins were identified in plasma, as outlined above, only nine SASP proteins (Table S4) in urine were age‐associated from the SASPAtlas (Basisty et al. 2020), including CTSD, MMP3, and fructose 1,6‐bisphosphatase 1 (FBP1). While none of the plasma age‐associated CTSs were age‐associated in urine, CTSD which was not age‐associated in plasma was over‐represented with age in urine. CTSD activates the PI3K/AKT/mTOR pathway (Yu et al. 2021), which plays a role in angiogenesis and endothelial cell senescence, and its increase is consistent with its association with the age‐associated decline in eGFR in our participants (Limonte et al. 2022; Carlsson et al. 2017). The rate‐limiting gluconeogenic FBP1 loss has been shown to induce senescence and SASP in hepatic stellate cells (Li et al. 2020). The over‐representation of MMP3 is likely a reflection of the involvement of MMPs in ECM degradation (Liang et al. 2021).

Analogous to our observation in plasma, many age‐associated proteins in urine were also ECM and ECM‐related (22 proteins (Table S6))—including ICAM2, ITGB6, SDC1, KDR, AMPB, and MEGF9—all consistent with a decline in kidney function that occurs with age. For example, AMPB, a housekeeping protein that plays a role in antioxidant defense, including reducing oxidation products formed on ECM structures and lipid peroxidation of cell membranes (Bergwik et al. 2021), was over‐represented with age, suggesting a decline in reabsorption by the tubular tissues with age, as high levels have been associated with interstitial fibrosis, tubular atrophy, and inflammation (Liao et al. 2018; Bakun et al. 2014). MEGF9, which was under‐represented with age in both plasma and urine, is involved in tissue repair (Brandt‐Bohne et al. 2007). ANGPTL1, over‐represented in plasma and urine, associates with ECMs, as mentioned above (Thorin et al. 2023). The differences in ITGB6, CAMs, and ECM components are consistent with a previous study on healthy aging, where the urine proteomes also identified ECM alterations and immune system dysfunction (Bakun et al. 2014).

A decline in renal function was also confirmed in this healthy population when considering age‐associated proteins (46) that were differentially expressed in plasma (34 over‐represented; 12 under‐represented) and urine (20 over‐represented; 26 under‐represented) at baseline (Table S9). While the majority of proteins changed in the same direction (28 proteins), 18 changed in opposite directions; 2 proteins' lower abundance in plasma: CA1 and GP1BA, suggests an age‐associated decline of re‐absorptive capacity (CA1) (Backman et al. 1990); and 16 proteins' higher abundance in plasma and decrease in urine suggest reduced clearance. The increasing plasma levels of CST3 (Hojs et al. 2006), HAVCR1 (Song et al. 2019), FGF23 (O'Sullivan et al. 2017), MB (also increasing in urine), and LCN2 (Moaddel et al. 2023) in the cross‐sectional analysis further support a decline in renal function. Of note, although previous studies suggested that renin levels decrease with age (Abadir 2011; Saravi et al. 2021), in contrast, in our study, the plasma levels of renin increased with age. However, this observation should be considered with caution because the proteomic platform used in this study does not distinguish between active‐ and pro‐renin.

### 2.4 Multivariate Signature of Aging

Conceptually, an individual's biological age is defined by the level of age‐dependent biological changes, such as molecular and cellular damage accumulation (Moqri et al. 2023). This is typically summarized as a number (in units of time) matching the chronological age where the average person in a reference population shares the individual's level of age‐dependent biological changes. The “aging clocks” are algorithms derived from measurements at the clinical, phenotypical, cellular, and molecular levels, aimed at estimating biological age (Rutledge et al. 2022). Previous work has shown that age acceleration (also known as age deviation), defined as the difference between an individual's biological and chronological age, can be associated with mortality, the onset of age‐associated chronic diseases, and frailty phenotypes (Moqri et al. 2023; Rutledge et al. 2022). This work, however, has been typically limited to blood samples, and most of it focused on epigenetic biomarkers, for which no causal or mechanistic hypotheses have been formulated (Hannum et al. 2013; Horvath 2013; Levine et al. 2018; Lu et al. 2019; Belsky et al. 2022).

In the healthy population of our study, we aimed to identify a set of predictors that, in weighted aggregates, predicts chronological age for each physiological compartment. Within the context of the aging clock paradigm, we hypothesized that deviation of these predictors from chronological age would associate with health dimensions. We also hypothesized that although the age biomarkers estimated in different compartments would include different molecules, independent of chronological age, they would be related because they each express the pace of chronological aging. To this end, we carried out cross‐validated elastic net regression models using plasma, urine, and muscle (Ubaida‐Mohien et al. 2019) proteomic data, in addition to the plasma, urine, and muscle metabolomic data (Moaddel et al. 2023) from the baseline study visit, to create a multivariate signature for each compartment/−omic as a predictor of chronological age.

Figure 2 shows the elastic net mixing parameter alpha and penalty parameter lambda selected in each compartment/omics (see Materials and Methods section for details), as well as Pearson's correlation between true chronological age (the response variable, shown along the x‐axis) and the out‐of‐bag predicted age (mean and standard deviation, displayed along the y‐axis). Based on deviations between predicted and the expected chronological age based on the full cohort, we determined aging scores for each individual in each one of the six compartment/−omic models. The proteins and metabolites retained in the global biomarkers are listed in Tables 2 and S10, which present molecular signatures for each compartment based on regression coefficient and frequency as two complementary metrics to assess feature significance (see Materials and Methods section for details). The majority of the proteins and metabolites in the signatures have been described in the cross‐sectional analysis (Tables S1, S7 and Ubaida‐Mohien et al. 2019; Moaddel et al. 2023) while those not identified are listed in Table S11. In plasma, the top associated metabolites (*p* < 0.05) were similar to the top age‐associated metabolites in the cross‐sectional analysis (Table S1), with only seven metabolites (0.05 < *p* < 0.10), including three tryiglycerides (TGs), three phosphatidylcholines (PCs), and cholesterol ester (CE) 15:0 newly identified. Several of the TGs and PCs contained Ω‐6 fatty acid and/or palmitic and oleic acids suggesting a healthy diet in this healthy population subgroup (oleic and linoleic acid consumption; Listenberger et al. 2003; Kien et al. 2005). Accordingly, an increase in pentadecanoic acid (C15:0) suggests an increase in the intake of milk products and fat from milk products (Smedman et al. 1999). Of interest, C15:0 is an AMPK activator and has anti‐inflammatory, anticancer, antifibrotic, antimicrobial, and mTOR‐inhibiting activities (Venn‐Watson and Schork 2023). It is believed to support cardiometabolic, immune, and hepatic health, with low circulating levels associated with increased risk of developing type 2 diabetes, heart disease, and nonalcoholic fatty liver disease (Venn‐Watson and Schork 2023). In urine, only citrulline was newly identified by the multivariate approach, consistent with the age‐associated difference observed in plasma in the cross‐sectional analysis (Moaddel et al. 2023). In the proteomic multivariate analysis, the top proteins in plasma, urine, and muscle were similar to the top age‐associated proteins identified in the cross‐sectional analysis (Tables S1, S7 and Ubaida‐Mohien et al. 2019). Moreover, many SASP proteins were also identified in the molecular signatures of plasma (13/73), muscle (23/47), and urine (5/75) (Table S4). Six proteins were newly identified in the multivariate analysis in plasma (Table S11): ECM protein MEPE, the cell adhesion protein MFGE8, the homophilic adhesion molecule CD99L2, the inflammatory cytokine IL1A, syndecan‐1 (SDC1), and advanced glycosylation end product‐specific receptor variant sRAGE2 (AGER). Consistent with our study, SDC1 was recently shown to decrease in plasma with age (Nam et al. 2021), and the identification of AGER is consistent with the age‐associated increases of AGEs and their association with increased risk of cardiovascular disease (Moaddel et al. 2021). Similarly, there were 26 newly identified proteins in urine (Table S11), predominantly classified as extracellular regions from the cellular component GO analysis and included several immune‐related proteins, including the CAMs VCAM1, CD99, and CD276, IL6, TNFSF13, lysosomal proteins IRAG2, DCN, and the ECM proteins CLC, COLEC12, CRTAC1, DCN, and SPP1. And in muscle, the top three identified proteins were also in the top five age‐associated proteins in the cross‐sectional analysis (Ubaida‐Mohien et al. 2019), with only prosaposin (PSAP), the precursor of lysosomal saposins that promote the degradation of glycosphingolipids newly identified: There were no newly identified metabolites in the muscle compartment compared to the cross‐sectional analysis (Moaddel et al. 2023).

**TABLE 2:** Multivariate signatures of chronological aging for different compartments (a) Plasma, muscle, urine proteomic signatures and (b) Plasma, muscle, urine metabolomic signatures obtained from cross‐validated elastic net models.

(a) Proteomics	
	Proteins
Plasma	LTBP2, CXCL17, GDF15, RET, WNT9A, EDA2R, ADAMTS16, CDON, PODXL2, CTSV, ITGAV, CD1C, NEFL, CRLF1, SCARF2, TNFRSF10B, IGFBP2, HSPB6, CDCP1, SOD2, RSPO3, CXCL9, SCARB2, PGF, NOMO1, MSR1, SMOC1, CCN5, TSPAN1, PCDH1, UXS1, FASLG, SNCG, PLAT, MSTN, CCDC80, IL17D, CDHR2, KIT, NTproBNP, IGFBP7, TREM2, RSPO1, TNFRSF11B, AGRP, FUT3_FUT5, CD99L2, FABP9, EGFR, LRRN1, OBP2B, MEPE, CRTAC1, CDHR1, IL1A, MEGF10, IGFBP1, SDC1, KITLG, MLN, CR2, GFAP, LGALS7_LGALS7B, MFGE8, SFRP1, SOST, METAP1, TNFSF11, F3, DCN, NFASC, CHIT1, AGER
Muscle	HNRNPA3, APCS, ALDH6A1, METAP2, TRPT1, WDR61, SSB, XRCC6, HNRNPU, ACADSB, FLNC, OXCT1, FBL, HSPB1, D2HGDH, NPM1, DBT, GLRX3, TPPP, PRPF19, GGCT, BCL2L13, FBLN5, SCARB2, HTATSF1, TPP1, SORD, SSPN, UBR3, EEF1B2, NCL, WDR77, PLEKHF1, HNRNPD, HNRNPUL2, MAPRE3, PSAP, FHL3, ABCE1, HCCS, MYBPH, UBQLN4, MUSTN1, MAP2K3, DNAJB5, LRRC47, SRSF3, ASAH1
Urine	CCN5, CD274, RGMA, TNFSF13, IL7R, MEGF9, CNTNAP2, TSPAN1, AMBP, LIF, CA4, CPA1, LAIR1, PI3, ITGB6, PAMR1, DSG4, TNFRSF13C, LIFR, ANGPTL1, FABP4, EDA2R, CRTAC1, TNFRSF21, REG3A, TCL1A, VCAM1, SERPINA12, SDC1, TNFRSF8, ADAM23, CALCA, OGN, IL6ST, TNFRSF10B, CLC, TNR, CD34, SCARF2, DKK4, CA1, OMD, CD244, CDSN, RSPO1, PPY, MLN, COLEC12, CLEC4D, NUDC, FHIT, IRAG2, BTC, PAEP, IL6, SPP1, PPP1R12A, PDCD1LG2, KAZALD1, AMBN, PODXL, DCN, FKBP4, FCER2, CTSD, ENPP5, CD99, CDHR2, GFRA3, TNFRSF9, CD276, CHIT1, DPEP1, TIMD4, GSTA1

(b) Metabolomics	
	Metabolites
Plasma	PC.aa.C40.6, Leu, CE.22.6, DHEAS, CE.15.0, Cystine, Ile, Cit, PC.aa.C34.4, TG.16.0_38.2, Cys, PC.aa.C40.4, TG.20.4_36.4, PC.ae.C36.4, TG.18.1_31.0.
Muscle	PC.aa.C36.4, Choline, HipAcid, PC.aa.C36.0
Urine	His, t4.OH.Pro, TG.16.1_38.4, Carnosine, Creatinine, DHEAS, Cit

*Note:* Features with negative coefficients are in blue (see Table S9 for details).

**FIGURE 3:**
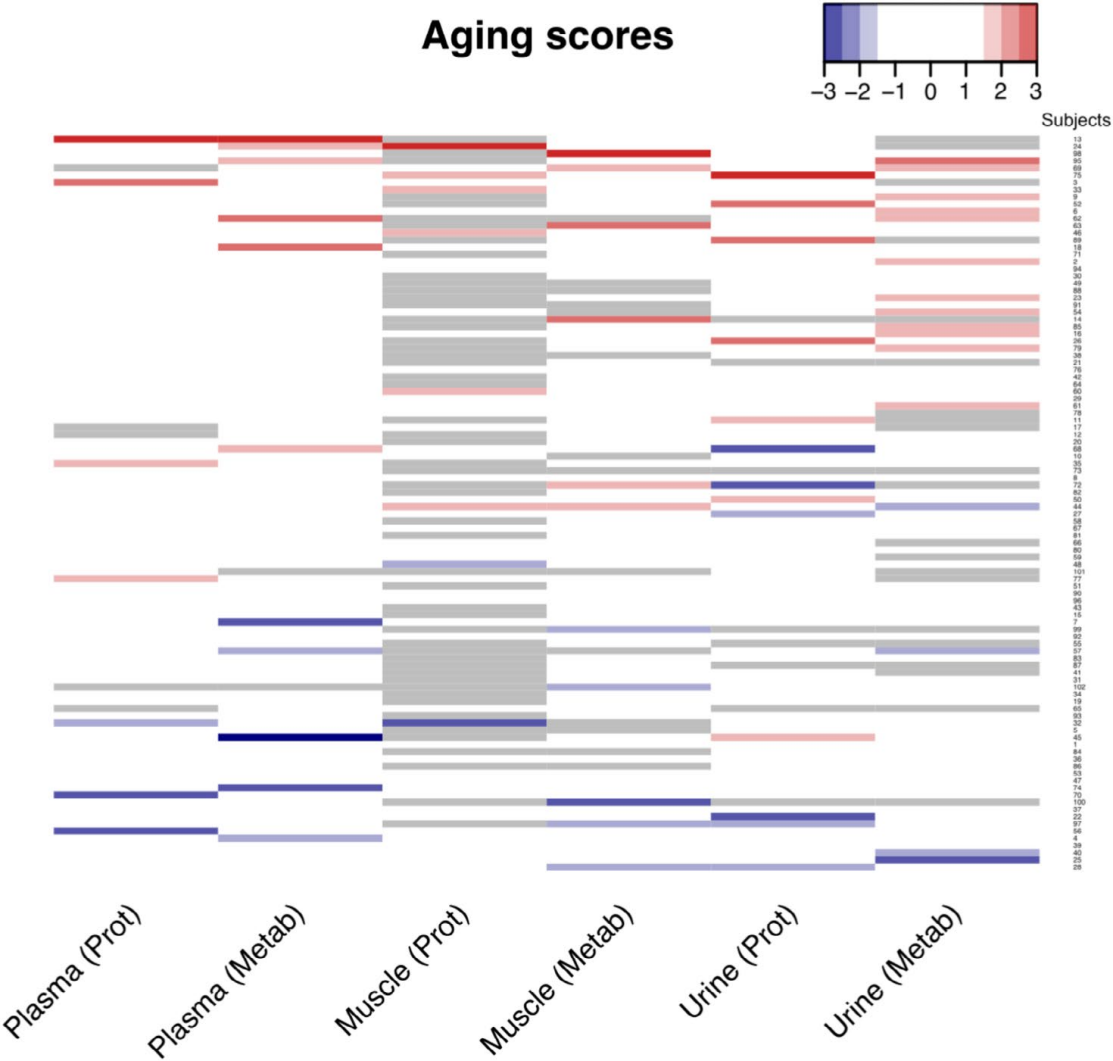
Aging scores across compartment/omics. Aging scores across compartment/omics (columns) and subjects (rows). After column‐wise *z*‐score transformation, significant deviations (|*z*| > 1.5) are shown in red for accelerated aging and blue for decelerated aging. Missing data are shown in gray.

A pairwise correlation matrix of age acceleration scores across six omics/compartments shows modest but significant correlations, despite different compositions in proteins and metabolites (Figure S4a). This observation, indeed, agrees with the fundamental tenets of the geroscience paradigm (Kennedy et al. 2014), namely, that biological aging is a fundamental process underlying a wide variety of manifestations at the phenotypical and functional levels, although recent evidence suggests that aging processes may selectively impact different tissues and organs in different individuals (Oh et al. 2023). We then averaged proteomic‐ and metabolomic‐derived scores within each compartment, and the pairwise correlation matrix of the age acceleration scores across the three compartments is shown in Figure S4b. Overall, these findings suggest that different cumulative scores reflect similar mechanisms for the deviation from healthy aging in spite of a very different set of biomarkers used to calculate them. Aging scores across compartments/−omics, with significant deviations (accelerated/decelerated) from the chronological age are illustrated in red/blue, respectively, in Figure 3, with a *z*‐score threshold of 1.5. Interestingly, in our study, the compartments deviated from chronological aging at different rates within a participant, consistent with other studies suggesting that aging rates are diverse across different compartments (Nie et al. 2022); however, in our study, the deviations from chronological age within a participant across compartments were predominantly in the same direction (Figure 3). We then selected the metabolites and proteins from our GESTALT plasma signature (Table S10) built from a generalized linear model to predict age using a leave‐one‐out cross‐validated (LOOCV) procedure and then calculated the correlation between the chronological age and the LOOCV predicted age (Figure S5). The metabolomic signature worked similarly well in the GESTALT study (*r* = 0.83, *p* = 2.4 e‐26) and in 162 participants from the Baltimore Longitudinal Study of Aging (BLSA) selected to have the same healthy characteristics of the GESTALT participants (*r* = 0.84, *p* = 1.4e‐43). Similarly, for the protein signature, we used the proteomic data from BLSA (*n* = 194 participants); however, in this case, proteomics was carried out using a different platform (1.3 k SomaScan; Tanaka et al. 2018; Candia et al. 2017) resulting in only 45 overlapping proteins. Despite different proteomic platforms, the resulting proteomic score predicted chronological age similarly well in GESTALT (*r* = 0.89, *p* = 2.4e‐33) and BLSA (*r* = 0.89, *p* = 7.3e‐68; Figure S5).

**FIGURE 4:**
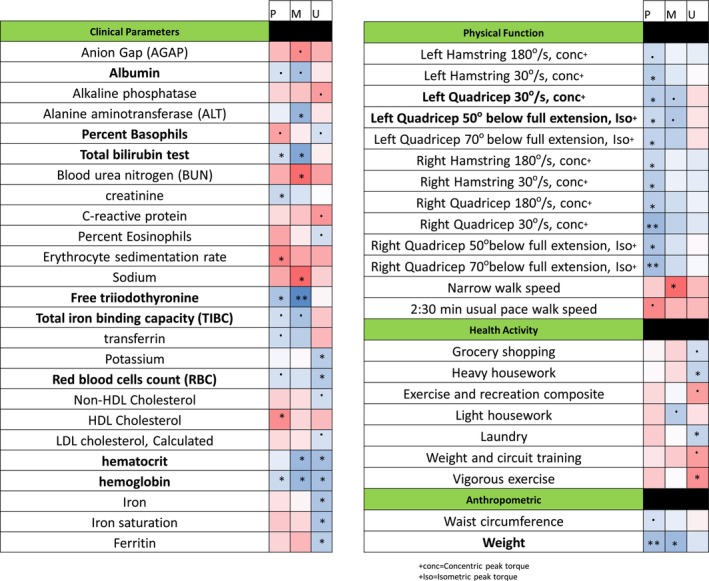
Heat map of clinical co‐variates associated with aging scores. Differences in clinical parameter, physical function, health activity and anthropometrics associated by spearman correlation (*p* < 0.10) with the aging scores based on the molecular signature from participants that had at least measurements in four of the six compartment/omics (*n* = 92) are reported. Bold indicates more than one compartment. Color indicates direction of association and significance is indicated (0.05 < *p* < 0.10; *0.01 < *p* < 0.05; **0.001 < *p* < 0.01; ***p < 0.001).

To improve the informative value of our aging scores, we created composite scores within each compartment by averaging the separate scores derived from protein‐ and metabolite‐based models. Considering only participants who had measurements in at least four of the six compartments/omics (*n* = 92), we then tested whether these compartment‐specific composite scores independently correlated with health variables that had been collected in the study, including clinical parameters, physical function, health activity, and anthropometrics. Spearman's correlation coefficients of accelerated age with clinical covariates with *p* < 0.10 are listed in Figure 4 and Table S12A, with clinical parameters with *p* > 0.10 listed in Table S12B. Differences in several of the clinical parameters were associated with deviations from chronological age, and the direction of associations was consistent with what would be expected with accelerated aging, demonstrating that the molecular signatures identified across compartments are predictive of accelerated aging. Hemoglobin levels were negatively associated in plasma, muscle, and urine (Figure 4), with eight other clinical parameters associated in two compartments (Figure 4). Low circulating albumin levels are associated with higher all‐cause mortality, cardiovascular disease, and other adverse outcomes (Kobayashi et al. 2023), and lower free T3 levels that were present with accelerated aging are consistent with reported decreases with aging (Taylor et al. 2023) and consistent with the role of T3 in maintenance of muscle (Ucci et al. 2019) and brain function (Bernal 2017). Several clinical parameters were associated with low iron levels in multiple compartments (lower hematocrit, TIBC, and RBCs) and one compartment (lower iron, iron saturation, transferrin, and ferritin levels). Considering that in the GESTALT study, anemia was an exclusion criterion at baseline, the association of hemoglobin with several clinical parameters and the predisposition to iron deficiency anemia (which at this stage was still not clinically evident based on normative clinical laboratory levels) appear to be related to global aging across multiple tissues. This interpretation is consistent with findings that anemia is one of the most constant risk factors for age‐related frailty (Ruan et al. 2019) and increased morbidity and mortality in older people (Lanier et al. 2018). A decline in hepatic function in the age‐accelerated group can be interpreted with the association of low levels of bilirubin and higher alkaline phosphatase with accelerated aging (Kim et al. 2015). Increased levels of alkaline phosphatase are consistent with human tissue aging and reflect tissue hardening and calcification. Similarly, the higher BUN levels and AGAP concur with declining renal function in the accelerated group. Increased inflammation is also suggested with increased age acceleration, as we did indeed find increases in CRP and erythrocyte sedimentation rate—both of which are classic hallmarks of inflammation. Several other differences are consistent with age acceleration including a decline of creatinine that is in keeping with decreased muscle mass. The higher levels of sodium in the accelerated group are consistent with its reported association with dehydration, chronic disease risk, and accelerated biological aging (Dmitrieva et al. 2023).

Muscle strength is important to healthy aging, and a decline in both hamstring and quadriceps volume and strength occur with aging (Palmer et al. 2017), with quadriceps being the most susceptible to age‐related muscle loss in the extremities (Fuchs et al. 2023). In our study, a decline in hamstring and quadriceps muscle strength was observed in the accelerated group across several metrics and across compartments (plasma and muscle). This is consistent with the decrease of creatinine, as mentioned above, we found in the accelerated group and the expected loss of muscle mass and physical function that occur with accelerated aging. For the physical performance metrics containing walking distance and/or speed, height was added as a covariate. A faster speed for the 6 m narrow walk was surprisingly associated with accelerated aging; however, while counterintuitive, an increase in narrow walk speed may reflect a compensatory, focused strategy to minimize stepping outside the narrow (20 cm) course and would therefore be consistent with accelerated aging. Further, these associations were only observed in one compartment.

Levels of physical activity were assessed from a modified version of the Minnesota Leisure Time Physical Activity Questionnaire (Moore et al. 2024; Taylor et al. 1978), which asked participants to self‐report incidental and volitional exercise activities in the 2 weeks prior to testing. Several of the incidental activity parameters had lower reported activity (kcal/kg/week) with accelerated aging including household chores, grocery shopping, housework, and laundry. A decline in weight and waist circumference was also observed in the accelerated aging group, consistent with the notion that unintentional weight loss in aging is a biomarker of declining health (Fabbri et al. 2015).

**FIGURE 5:**
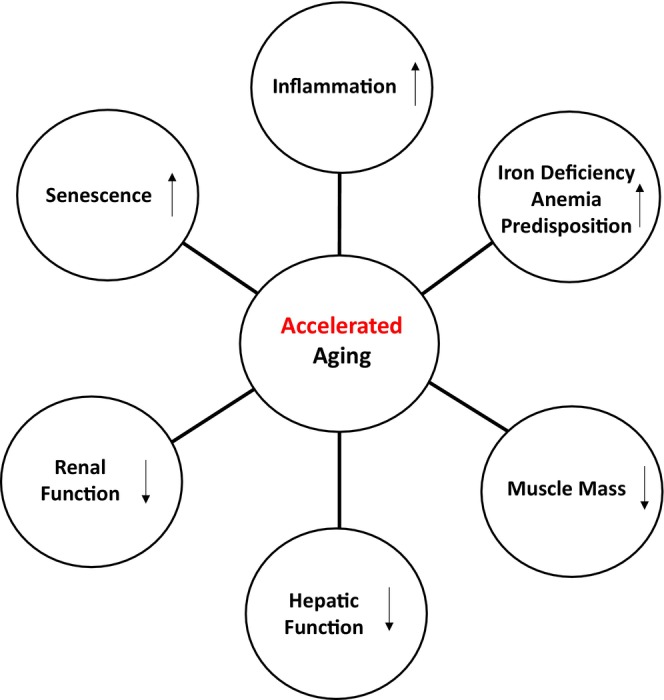
Summary scheme of the clinical phenotypes associated with accelerated aging using the molecular signatures from participants with measurements in at least four of the six compartments/omics.

Although a salient aspect of our study is that it enables investigation of multi‐omic signatures of healthy aging across multiple physiological compartments, one key limitation is the relatively small cohort size, which severely limits its statistical power and, therefore, its potential for discovery. As such, this study remains an important exploratory effort, which leverages a unique cohort and multi‐omic datasets that are invaluable for elaborating hypotheses on possible mechanisms underlying healthy aging, thus suggesting directions for future research. Because of the small cohort size, as well as the large number of clinical correlates that we investigated in association with compartment‐specific aging scores, we reported *p*‐values without multiple testing correction and relaxed the standard *p*‐value thresholds of significance. A summary of our findings is shown in Figure 5.

## 3 Discussion

Aging is a multifactorial process and involves numerous interconnected systems characterized by a loss in the ability to maintain homeostasis and resulting in the functional decline of cells, tissues, and whole organs (Santos et al. 2019). Given such extreme complexity, understanding the biology of aging is probably one of the most challenging fundamental conundrums that scientists are currently addressing. Studies performed on single molecular pathways have contributed substantially to our understanding of aging, but the findings are difficult to connect in a unique biological aging paradigm. The availability of “‐omic biomarkers” that allow measurement of hundreds or thousands of molecules across multiple body compartments may help to identify key biological processes that occur with aging and potentially distinguish individuals that remain clinically healthy into late life. To identify phenotypical changes more reflective of healthy aging, individuals enrolled in this study were free of chronic diseases, allowing for a more detailed picture of healthy aging that could help identify differences between chronological and biological aging within and between compartments. Using combined ‐omics data, we developed a molecular signature correlated with chronological age and identified individuals in which the pace of compartment aging was either accelerated or decelerated. The metabolomic and proteomic differences in each compartment identified several age‐related processes that were correlated with chronological age (Tables 2 and 3): inflammation, senescence, oxidative stress, extracellular proteins (ECM), diet and microbiome, protein homeostasis, autophagy, mitochondrial lysosomal, and renal health. Interestingly, many of these are confirmatory of metabolomic changes we previously described in the same cohort (Moaddel et al. 2023). The changes in proteins and metabolites associated with aging may reflect either causal or compensatory processes; however, at this stage, the distinction between these processes remain largely hypothetical. The clinical covariates linked to accelerated aging also indicated increased inflammation, a strong predisposition to iron deficiency anemia, declines in muscle mass, as well as kidney and liver function. It is important to note that the cross‐sectional design of the study, which enrolled “healthy” individuals, may help reduce potential bias stemming from chronic diseases that are more prevalent in older populations. Conversely, this approach could introduce bias, as the “healthy” younger individuals selected may not necessarily maintain their healthy status as they age.

**TABLE 3:** Related pathways for metabolites identified in the molecular signatures in plasma (p), muscle (m), and urine (u).

Inflammation	Oxidative stress	Senescence	Mitochondrial health	Vascular health	Diet and microbiome	Renal health
DHEAS (p, u)	Cystine (p)	Choline (m)	Citrulline (p)	Citrulline (p, u)	Choline (m)	Choline (m)
Cystine (p)	Histidine (u)	PCs (p, m)	Ile (p)	Cholesterol esters (p)	Hippuric acid (m)	Histidine (u)
TGs (p, u)	Carnosine (u)		Leu (p)	(CE 15:0, CE 22:6)	TGs (p, u)	Creatinine (u)
Choline (m)	Trans‐4‐hydroxyproline (u)		Trans‐4‐hydroxyproline (u)			
Histidine (u)	Hippuric acid (m)		Choline (m)			
Carnosine (u)	Ctrulline (p, u)		Histidine (u)			
Trans‐4‐hydroxyproline (u)	Choline (m)					
Hippuric acid (p)	Cholesterol esters (p)					
Citrulline (p, u)	(CE 15:0, CE 22:6)					

Perhaps the most interesting finding of our study was that one of the most enriched terms in every compartment in both the cross‐sectional and multivariate analysis was ECM (104, 100, and 28 proteins in plasma, muscle, and urine, respectively; Table S6). ECMs play a crucial role in maintaining tissue homeostasis (Blokland et al. 2020; Levi et al. 2020) by providing an external environment that helps mediate cellular activities (Sudevan et al. 2019) and because of their roles in regulating inflammatory responses (Bakun et al. 2014; Riley and Bradshaw 2020; Sorokin 2010), cellular senescence (Tominaga and Suzuki 2019; Levi et al. 2020) mitochondrial dynamics (Chen 2021), autophagy (Schaefer and Dikic 2021), and lysosomal health (Tancini et al. 2020; Coffey et al. 2021). Increased inflammation with age and age acceleration was observed with the change in proteins, metabolites (Moaddel et al. 2023), and/or clinical parameters (CRP and erythrocyte sedimentation rate, for example). Inflammation results in endoplasmic reticulum (ER) stress (Hasnain et al. 2012), which disrupts proteostasis, as one‐third of the proteome is synthesized in the ER (Martínez et al. 2017). While typically remarkable changes are not observed for ER in older age (Gil‐Hernandez and Silva‐Palacios 2020), in our study, ER age‐associated proteins were found to be present across compartments in plasma (71), urine (3), and muscle (197) (Ubaida‐Mohien et al. 2019) in both the cross‐sectional (Table S13) and multivariate analyses. Inter‐organelle contact sites play important roles as regulators of cellular homeostasis (Peng et al. 2020). The interactions of ER and mitochondria form mitochondria‐associated ER membranes (MAMs) (Degechisa et al. 2022), which, under chronic ER stress, result in mitochondrial dysfunction that contributes to the progression of age‐related diseases (Gil‐Hernandez and Silva‐Palacios 2020). Several MAM proteins were age‐associated in muscle (Ubaida‐Mohien et al. 2019); protein disulfide isomerases (PDIA3, PDIA4, PDIA6, PDIA1), MCU, a regulator of senescence (Gil‐Hernandez and Silva‐Palacios 2020), DRP1 (UTRO), AKT, PP2A (PP2AA), RRBP1, and TMX1, CKAP4 increasing and ERO1α decreasing in muscle (Gil‐Hernandez and Silva‐Palacios 2020; Kumar and Maity 2021), and CKAP4 also increasing in the cross‐sectional analysis in plasma. These proteins have multiple functions such as mitochondrial dynamics and morphology (CKAP, DRP1), metabolism (AKT, PP2A), redox state (ERO1α), and MAM tethering (RRBP1) (Gil‐Hernandez and Silva‐Palacios 2020; Kumar and Maity 2021). The role of these proteins should be further examined in relationship with mitochondrial functions in multiple human tissues.

Inter‐organelle contact sites between mitochondria and lysosomes have also been characterized (Peng et al. 2020). In our study, 34 lysosome proteins in plasma including 6 CTSs and 105 proteins (Table S14) in muscle (Ubaida‐Mohien et al. 2019), including 2 CTSs, were age‐associated from the cross‐sectional analysis, with CTSV and CTSD also identified in the molecular signatures in plasma and urine, respectively, suggesting greater lysosomal membrane permeabilization (Moaddel et al. 2023). The neutral ceramidase (ASAH2) and the lysosome membrane protein 2 (LIMP2 (Scarb2)) were also age‐associated in plasma, similar to our previous study (Tanaka et al. 2018), with LIMP2 and acid ceramidase (ASAH1) identified in the molecular signature of plasma and muscle, respectively. Changes observed in ER–mitochondria interactions, mitochondria health, lysosomal dysfunction, and ECM components, which all play a central role in regulating cellular senescence (Tominaga and Suzuki 2019; Huang et al. 2022), suggest an important link between senescence, aging, and age‐related pathologies, consistent with the large number of age‐associated proteins in our study (Table S4) identified by the SASPAtlas (Basisty et al. 2020).

In tissues and organs, the ECM forms intricate networks that provide a physical scaffold for cells and overall structural support, compressive strength, and elasticity in all tissues and organs (Arriazu et al. 2014). In this study, a decline in muscle strength and hepatic and kidney functions was observed in the accelerated aging group. These are consistent with ECM changes, as the ECM serves as a scaffold for muscle cells to attach, proliferate, and transform in response to muscle injury or disease (Ahmad et al. 2023); has been shown to regulate the induction of alkaline phosphatase in fibroblasts (Abe et al. 2001); and high accumulation of ECMs is anathema to renal function by compromising the cell‐total nephron‐ECM crosstalk (Kim et al. 2022). Several clinical parameters (BUN, AGAP), as well as proteomic and metabolomic changes, are reflective of declining renal function. The kidney is necessary for the clearance of many circulating proteins and peptides (Shrestha et al. 2018), with levels of circulating proteins regulated by production and secretion, followed by filtration through nephrons and subsequent re‐absorption or clearance. The accumulation of anatomic and functional damage in the kidney with age‐coupled with a finite number of irreplaceable nephrons, resulting from oxidative stress, inflammation, and alternations in calcium regulation and the renin–angiotensin system (RAS) (Jang et al. 2018), can lead to progressive decreases in GFR, consistent with our study (Moaddel et al. 2023).

## 4 Conclusions

The data in this study are consistent with significant alterations in ECM composition and integrity with age across compartments. The data support the notion that interventions targeting ECM homeostasis may slow the rate of age‐related dysfunction. Further, we developed metabolomic and proteomic molecular signatures for each compartment that correlate with clinical parameters, suggesting that these multi‐omic indices may capture accelerated aging. The overall strengths of this study include validation of the findings in multiple cohorts and the study across multiple compartments within the same individual. Limitations include that the proteomic assessment of plasma and urine was carried out by PEA, while the muscle proteomic data was carried out by LC–MS/MS, limiting the potential identification of cross compartment changes; the exploratory nature of the study is due to small sample size, which consequently did not permit evaluation of sex‐ and race‐specific associations. While measuring additional tissues would likely add insights into the mechanism of age‐related differences, we are limited to tissues that are accessible from human participants in studies such as ours.

## 5 Materials and Methods

### 5.1 Study Design and Participants

Fasting plasma samples from baseline (*n*_participants_ = 101) and from 2‐year follow‐up (*n*_participants_ = 65), and urine from baseline (*n*_participants_ = 95) and from 2‐year follow‐up (*n*_participants_ = 65), analyzed in this study were collected from participants from the Genetic and Epigenetic Study of Aging and Laboratory Testing (GESTALT). Screening medical exam and blood tests were evaluated to determine whether a candidate participant met inclusion criteria including: free of major diseases, except for controlled hypertension or a history of cancer that had been clinically silent for at least 10 years, no chronic medication use (except one antihypertensive drug), no physical or cognitive impairment, and BMI less than 30 kg/m^2^. Inclusion criteria were gathered from information on medical history, physical exams, and blood tests interpreted by a trained nurse practitioner at screening and the first study visit (Ubaida‐Mohien et al. 2019; Tanaka et al. 2018). A full list of inclusion criteria at the baseline visit is provided in Table S15. Participants were evaluated at the Clinical Research Unit of the National Institute on Aging Intramural Research Program. Whole blood samples were collected using BD vacutainer tubes with ethylenediaminetetraacetic acid (EDTA), then centrifuged at 2300 rpm at 20°C for 15 min; plasma was separated into 500 μL aliquots and stored at −80°C until assay. Participants provided fasting urine (8 h minimum) samples into a 120 mL sterile urine collection container. Upon collection, the urine was pipetted into 925 μL aliquots. Both plasma and urine aliquots were placed at −80°C until assay.

### 5.2 Proximity Extension Assay Proteomic Analysis

Proteins were measured using Olink Explore [state 1536] (Olink Proteomics AB, Uppsala, Sweden) according to the manufacturer's instructions. The technology behind the Olink protocol is based on Proximity Extension Assay (PEA) (Assarsson et al. 2014), coupled with readout via next‐generation sequencing (NGS). The assay enables detection of up to 1536 proteins in 90 samples simultaneously, using only 2.8 μL of serum/plasma. In brief, pairs of oligonucleotide‐labeled antibody probes designed for each protein bind to their target, bringing the complementary oligonucleotides in close proximity and allowing for their hybridization. The addition of a DNA polymerase leads to the extension of the hybridized oligonucleotides, generating a unique protein identification “barcode”. Next, library preparation adds sample identification indexes and the required nucleotides for Illumina sequencing. Prior to sequencing using the Illumina NovaSeq 6000, libraries go through a bead‐based purification step, and the quality is assessed using the Agilent 2100 Bioanalyzer (Agilent Technologies, Palo Alto, CA). The raw count data was generated using bcl2counts (v2.2.0) and was quality‐controlled, normalized, and converted into Normalized Protein eXpression (NPX), Olink's proprietary unit of relative abundance. Data normalization is performed using an internal extension control and an external plate control to adjust for intra‐ and inter‐run variation. All assay validation data (detection limits, intra‐ and inter‐assay precision data, predefined values, etc.) are available on the manufacturer's website (www.olink.com).

### 5.3 Clinical Measurements

A clinical exam and anthropometric measurements are completed by a research‐trained nurse using a series of standardized protocols. Demographic and health behavior information are assessed through a structured interview. In addition to traditional clinical evaluations, GESTALT participants complete a wide range of physical performance testing as previously described (Kuo et al. 2020). All measurements are made by highly trained study staff using standardized protocols. Clinical assays are performed in the Clinical Laboratory Improvement Amendments (CLIA) certified clinical laboratory of Harbor Hospital (Baltimore, Maryland).

### 5.4 Data Processing, Statistical Analysis, Data Modeling, and Visualization

Processed, quality control‐normalized proteomic data were log‐transformed, and only proteins with > 70% below the limit of detection in samples were excluded from analysis. Principal component analysis was used for multivariate statistics, specifically for outlier detection, compartment differences, and visualization of multiple compartments.1432 and 1427 proteins were filtered out for 101 plasma and 95 urine proteomic samples. Proteins that had assays deviating from negative controls (*z*‐score) greater than +4SD from the *z*‐score mean were excluded prior to analysis. Each protein was analyzed for age association by a multivariable linear regression model adjusted for sex, race, and BMI using the lm() function from the base R package stats (v. 4.2.3). To account for multiple comparisons, *p*‐values were corrected by the Benjamini–Hochberg procedure to control the false discovery rate and reported in Tables S1 and S7. The threshold for statistical significance was *p*‐value < 0.05 unless otherwise reported for proteins. Calculated p‐values for age association and effect size (*β*_age_ or log2 fold‐change per year) are indicated in the figure panels or legends. All statistical analysis were conducted using R (4.2.3) with built‐in libraries and functions. The data processing for plasma, urine, and muscle metabolomics and skeletal muscle proteomics follow more or less the same analytical pipeline, and the details were reported here (Ubaida‐Mohien et al. 2019; Moaddel et al. 2023).

We conducted a comparison of our cross‐sectional analysis carried out by proximity extension assay (PEA) and a cross‐sectional analysis that used an aptamer‐based proteomic assay (SOMA scan) in 162 participants of the Baltimore Longitudinal Study of Aging (BLSA) selected to have the same health status characteristics of the GESTALT study (see Materials and Methods section; Tanaka et al. 2018). Of the 553 common proteins in both studies, 161 were age‐associated in both studies with a strong correlation (*r* ^2^ = 0.65, *p* = 2.640e‐28; Table S3B), and 205 were not age‐associated in either study.

In order to build multivariate, omics‐based predictors of chronological age, we used the elastic net (Zou and Hastie 2005), which is a regularized generalized linear modeling and variable selection method that offers flexibility and accounts for multi‐collinearity in the predictor variables. The elastic net depends on two hyperparameters: the penalty parameter lambda, which defines variable shrinkage, and the mixing parameter alpha, which can be continuously tuned between ridge (alpha = 0) and lasso (alpha = 1) regression. To select the optimal hyperparameter values, we used eNetXplorer (Candia and Tsang 2019), an R package that implements a cross‐validated approach and generates model significance and predictive scores. To prevent random chance results, eNetXplorer implements a hierarchical cross‐validation algorithm. First, at the model level, it compares the cross‐validated model against an ensemble of cross‐validated null models generated from random permutations of the response. Model performance is quantified by a quality function (in our case, Pearson's correlation); the comparison between the model's performance and the null models' performance distribution is quantified by an empirical *p*‐value. Second, at the feature level, a similar procedure is used to quantify the performance of each feature in the model against that feature's performance across the null model ensemble. To capture feature‐level performance, two metrics are used, the regression coefficients and the frequency associated with each feature being chosen in the regularized regression procedure. This framework has recently been applied to transcriptomics (Candia et al. 2020), systems immunology (Sparks et al. 2023), brain imaging (Pat et al. 2023), serum proteomics (Minas et al. 2022), and brain proteomics (Roberts et al. 2023) data, among others, showing the ability to scan a high‐dimensional feature space to find cross‐validated signatures predictive of a clinical outcome. In our study, these models provide an optimal, cross‐validated prediction of age based on the simultaneous assessment of a large number of variables, including the metabolomic and proteomic data from plasma, muscle, and urine. The main outcomes are: (i) a multivariable signature of features that, when taken together, are optimal predictors of age; and (ii) aging scores, which measure departures (in either direction) between an individual's predicted age and the expected predicted age for that individual based on the full cohort. For each compartment/omics model, the number of samples (*n*) and protein/metabolite measurements (*p*) analyzed were as follows: *n* = 97, *p* = 1432 (plasma proteomics); *n* = 100, *p* = 435 (plasma metabolomics; Moaddel et al. 2023); *n* = 50, *p* = 2516 (muscle proteomics; Ubaida‐Mohien et al. 2019); *n* = 88, *p* = 144 (muscle metabolomics; Moaddel et al. 2023); *n* = 94, *p* = 1055 (urine proteomics); and *n* = 81, *p* = 94 (urine metabolomics; Moaddel et al. 2023). Sex was included in all models as covariate. For each model, 500 eNetXplorer runs were performed using fivefold cross‐validation and 125 null‐model permutations per run.

For the plasma and urine cross‐sectional analysis, STRING analysis (STRING: functional protein association network (https://string‐db.org/)) was carried out using the full STRING network, active interaction sources (Textmining, experiments, databases, co‐expression, neighborhood), and prediction with highest confidence (0.900).

## Author Contributions

R.M. and L.F. planned and interpreted the overall studies. R.M., M.Z., G.F., J.F., S.D., E.L. contributed to the proximity extension assay experiments. G.F., N.S., M.K. collected and secured clinical samples. T.T., A.Z.M., E.S., S.C., J.D. collected the clinical covariate data. R.M., J.C., C.U.‐M. performed data analysis. R.M., J.C., C.U.‐M. prepared the figures and tables. R.M. wrote the original article with critical review from J.M.E. and L.F. All authors contributed to editing the final version of the manuscript.

## Conflicts of Interest

The authors declare no conflicts of interest.

## Software Availability

Elastic net models were implemented via the R package eNetXplorer (Candia and Tsang 2019) version 1.1.3, publicly available at https://github.com/juliancandia/eNetXplorer.

## Supporting information


**Figure S1.** Age beta correlation between baseline and 2‐year follow‐up. (a) All plasma proteins from baseline and the 2‐year follow‐up are correlated to each other. (b) All urine proteins from baseline and the 2‐year follow‐up are correlated to each other.

**Figure S2.** Network representation of age‐associated proteins in plasma. (a) The STRING network analysis of the 390 over‐represented age‐associated proteins and (b) 85 under‐represented age‐associated proteins at baseline in GESTALT participants. (c) Identification of GO immune system process proteins within the over‐represented age‐associated protein network. STRING analysis for proteins using the full STRING network, active interaction sources (Textmining, experiments, databases, co‐expression, and neighborhood), and prediction with the highest confidence (0.900).

**Figure S3.** Urine age beta correlation between (a) correlation of Cystatin C levels in urine with age. (b) Age‐associated proteins in urine using a linear regression model adjusted for sex, race, and body mass index (BMI) and those also adjusted for urinary cystatin C and (c) for all proteins.

**Figure S4.** Pairwise correlation matrix of age acceleration scores. (a) Pairwise correlation matrix of age acceleration scores across six omics/compartments and (b) across three compartments with the averaged proteomic‐ and metabolomic‐derived scores within each compartment.

**Figure S5.** Validation of the plasma signatures in the BLSA cohort. Validation of the plasma metabolite signature (Table S10) in the GESTALT cohort (*n* = 100 participants) (A) and BLSA cohort (*n* = 162 participants selected to have the same health status characteristics of the GESTALT study). (B) Validation of the plasma protein signature (Table S10) in the GESTALT (C) and BLSA cohort (*n* = 194 participants selected to have the same health status characteristics of the GESTALT study). (D) BLSA proteomic data were obtained using the 1.3 k SomaScan proteomic platform, with only 45 proteins overlapping between platforms.

Table S1.

Table S2.

Table S3.

Table S4.

Table S5.

Table S6.

Table S7.

Table S8.

Table S9.

Table S10.

Table S11.

Table S12.

Table S13.

Table S14.

Table S15.

## Data Availability

All data associated with this study are available from the authors. Proteomic data have been provided as Tables [Link], [Link]. The source data underlying all presented figures and tables are provided as a Source Data file.
