# Supplementary figures and images for: Healthy Aging Metabolomic and Proteomic Signatures Across Multiple Physiological Compartments
Source: Aging Cell. 2025 Feb 14;24(6):e70014. doi: 10.1111/acel.70014 (PMC12151885; doi:10.1111/acel.70014)

Fig S1.

(a)

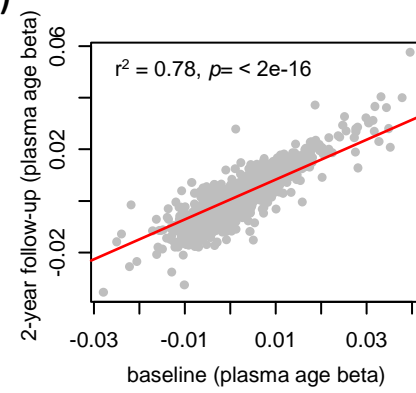

(b)

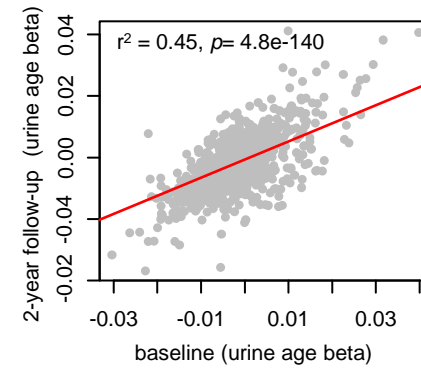

Supplement: Supplementary file 1 — Figure S1. Age beta correlation between baseline and 2‐year follow‐up. (a) All plasma proteins from baseline and the 2‐year follow‐up are correlated to each other. (b) All urine proteins from baseline and the 2‐year follow‐up are correlated to each other. [file ACEL-24-e70014-s002.pdf]

**a**

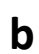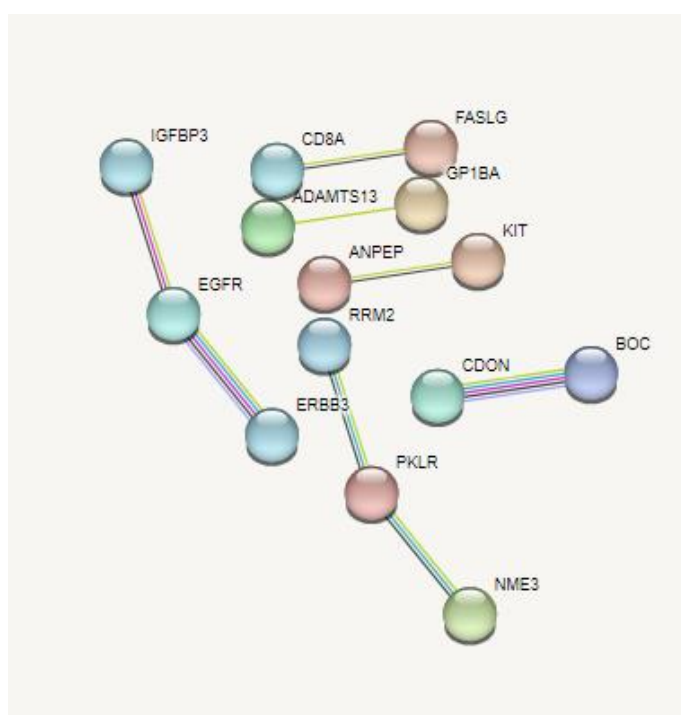

**C**

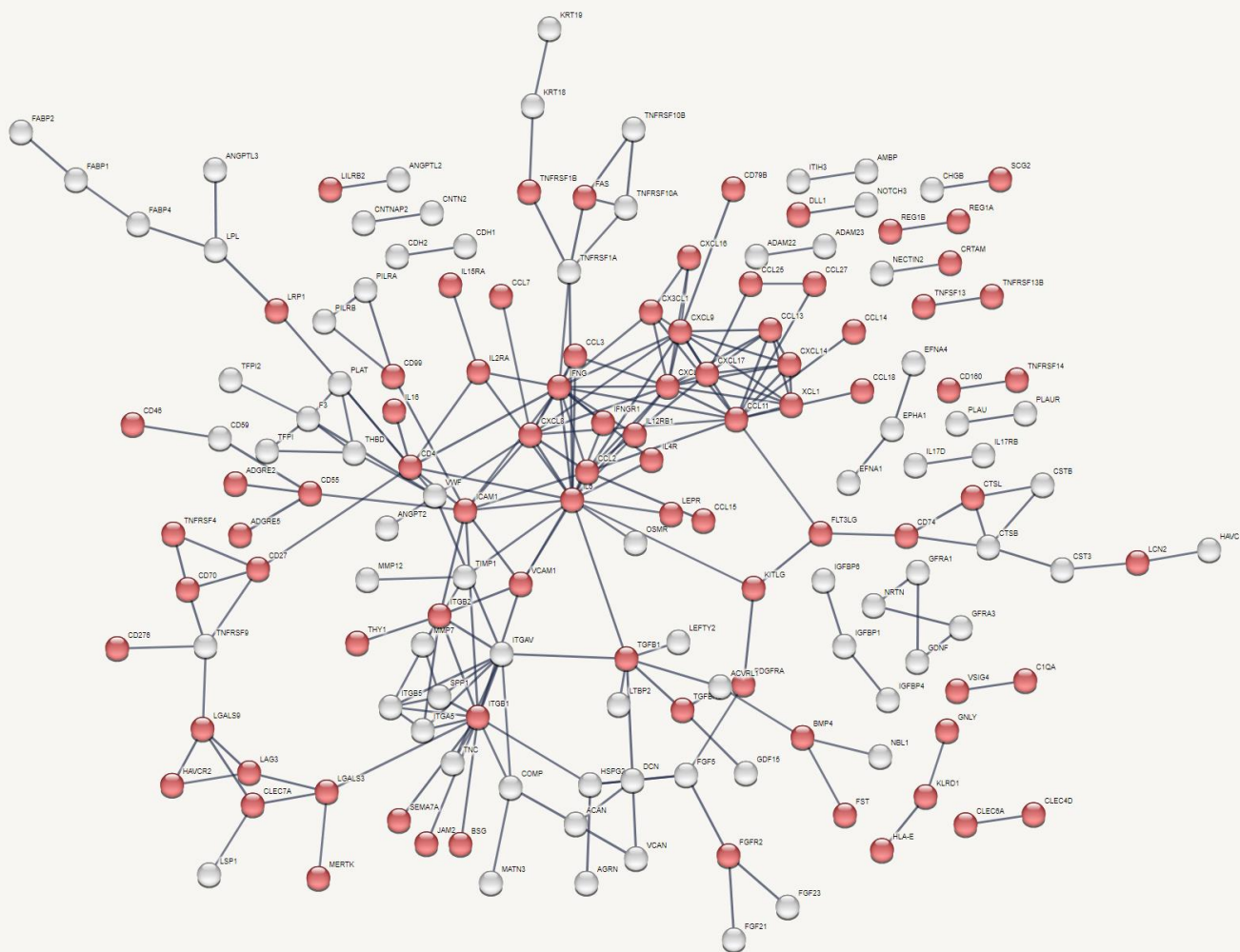

Supplement: Supplementary file 2 — Figure S2. Network representation of age‐associated proteins in plasma. (a) The STRING network analysis of the 390 over‐represented age‐associated proteins and (b) 85 under‐represented age‐associated proteins at baseline in GESTALT participants. (c) Identification of GO immune system process proteins within the over‐represented age‐associated protein network. STRING analysis for proteins using the full STRING network, active interaction sources (Textmining, experiments, databases, co‐expression, and neighborhood), and prediction with the highest confidence (0.900). [file ACEL-24-e70014-s020.pdf]

(a)

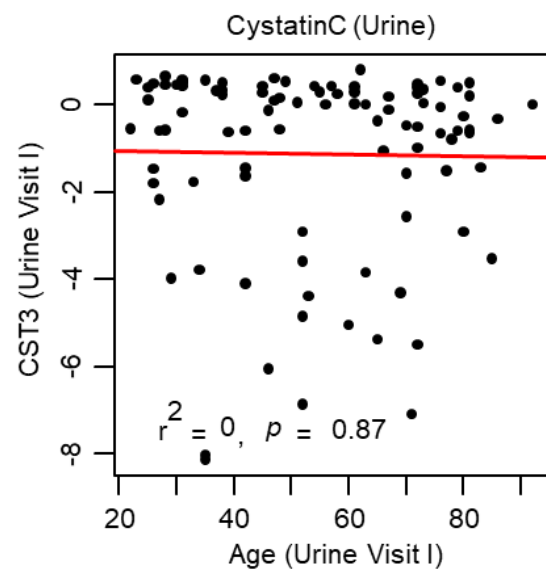

(b)

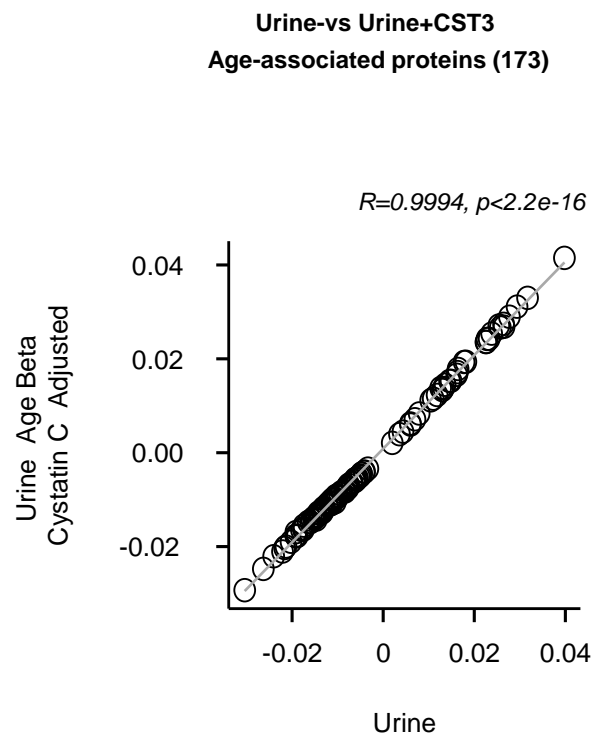

(c)

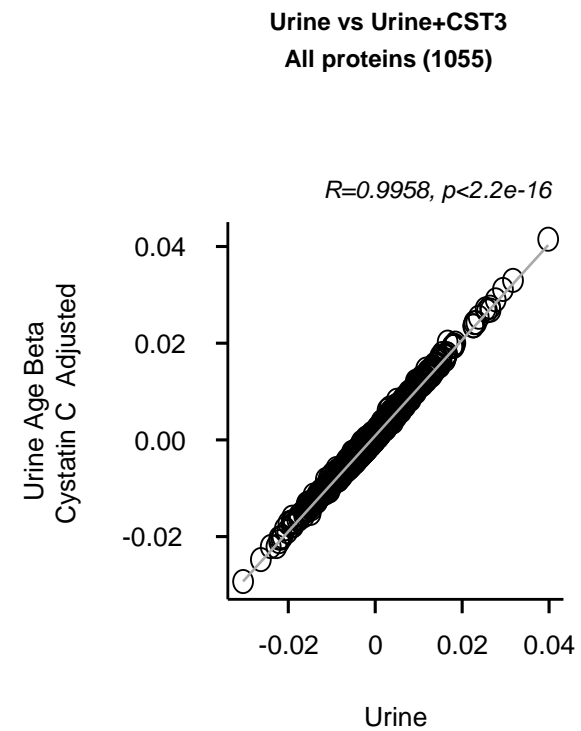

Supplement: Supplementary file 3 — Figure S3. Urine age beta correlation between (a) correlation of Cystatin C levels in urine with age. (b) Age‐associated proteins in urine using a linear regression model adjusted for sex, race, and body mass index (BMI) and those also adjusted for urinary cystatin C and (c) for all proteins. [file ACEL-24-e70014-s013.pdf]

**A** GESTALT (n=100)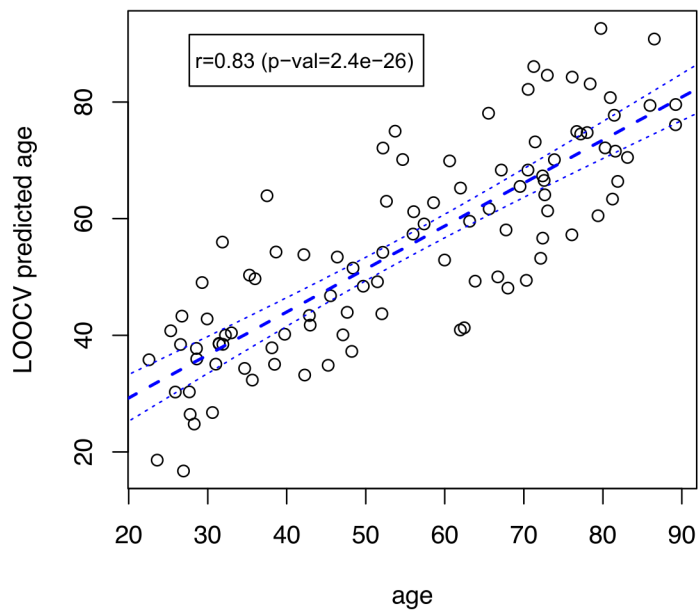**B** BLSA (n=162)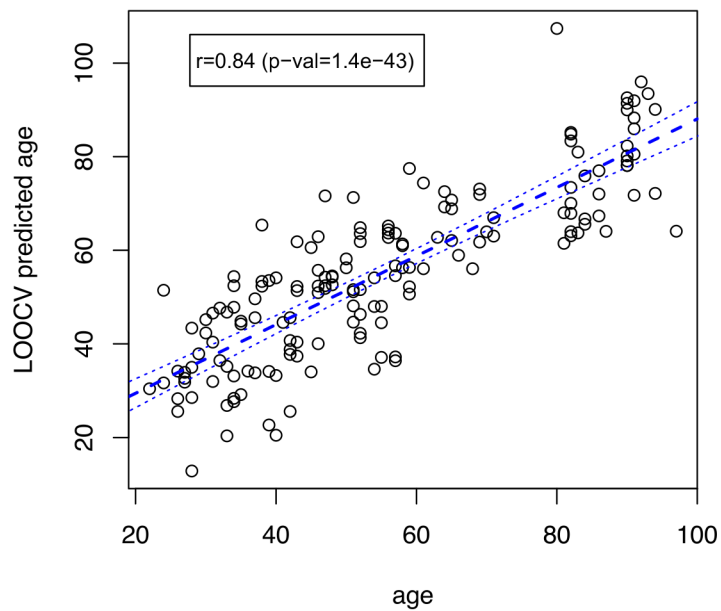**C** GESTALT (n=97)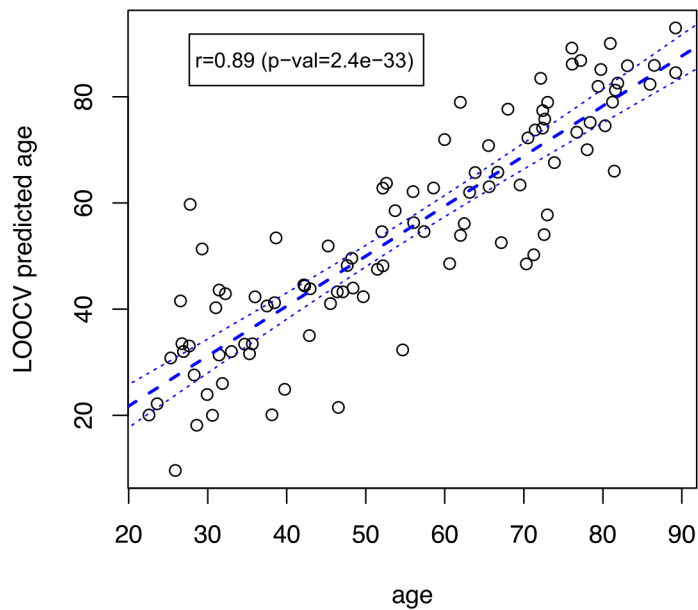**D** BLSA (n=194)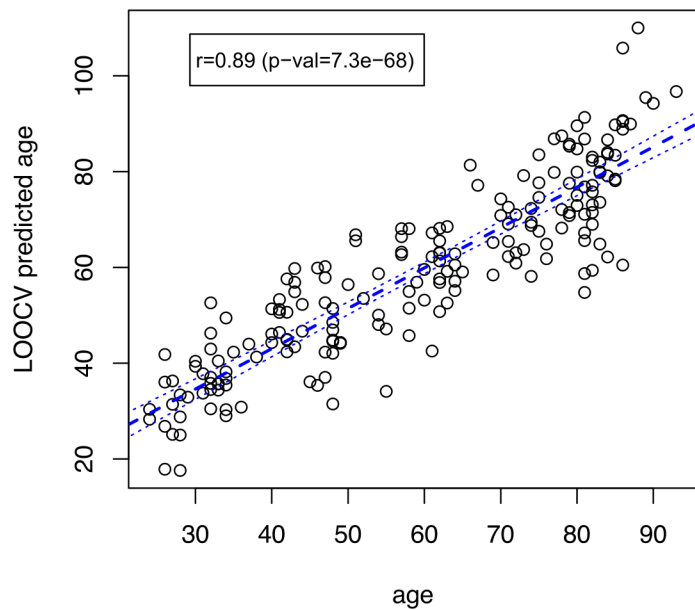

Supplement: Supplementary file 5 — Figure S5. Validation of the plasma signatures in the BLSA cohort. Validation of the plasma metabolite signature (Table S10) in the GESTALT cohort (n = 100 participants) (A) and BLSA cohort (n = 162 participants selected to have the same health status characteristics of the GESTALT study). (B) Validation of the plasma protein signature (Table S10) in the GESTALT (C) and BLSA cohort (n = 194 participants selected to have the same health status characteristics of the GESTALT study). (D) BLSA proteomic data were obtained using the 1.3 k SomaScan proteomic platform, with only 45 proteins overlapping between platforms. [file ACEL-24-e70014-s014.pdf]
