# Supplementary material for: Healthy Aging Metabolomic and Proteomic Signatures Across Multiple Physiological Compartments
Source: Aging Cell. 2025 Feb 14;24(6):e70014. doi: 10.1111/acel.70014 (PMC12151885; doi:10.1111/acel.70014)
Supplement: Supplementary file 4 — Figure S4. Pairwise correlation matrix of age acceleration scores. (a) Pairwise correlation matrix of age acceleration scores across six omics/compartments and (b) across three compartments with the averaged proteomic‐ and metabolomic‐derived scores within each compartment. [file ACEL-24-e70014-s007.pdf]

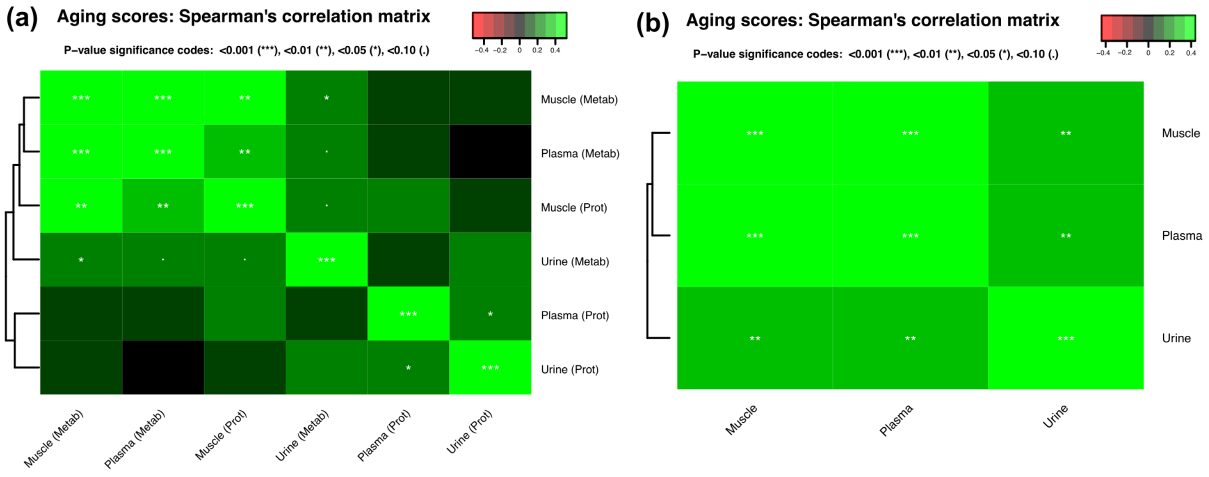

(a)
Aging scores: Spearman's correlation matrix
P–value significance codes: <0.001 (***), <0.01 (**), <0.05 (*), <0.10 (.)
−0.4 −0.2 0 0.2 0.4
Muscle (Metab)
Plasma (Metab)
Muscle (Prot)
Urine (Metab)
Plasma (Prot)
Urine (Prot)
(b)
Aging scores: Spearman's correlation matrix
P–value significance codes: <0.001 (***), <0.01 (**), <0.05 (*), <0.10 (.)
−0.4 −0.2 0 0.2 0.4
Muscle
Plasma
Urine
